# Middling whiteness: The shifting positionalities of Europeans in China

**DOI:** 10.1177/14687968211058014

**Published:** 2022-02-01

**Authors:** Aldina Camenisch

**Affiliations:** Amsterdam Institute for Social Science Research, 100440 University of Amsterdam, Amsterdam, Netherlands and nccr - on the move, University of Neuchâtel, Neuchâtel, Switzerland

**Keywords:** European migrants, positionality, racialisation, whiteness, middling migration, China

## Abstract

Drawing on ethnographic research, this article explores the multifaceted, situational and shifting social boundaries in the translocational positionalities of middle-class Swiss migrants in China. The analysis unpacks the central role of whiteness and Western ethnicity under the ‘Chinese gaze’, intersected by nationality. However, while they are marked by continuities of white privilege, the resulting racialised positionalities of the foreign white ‘others’ are characterised by a simultaneous elevation and subjugation, equally shaped by a ‘Chinese ascendancy’. European migrants’ self-positionings meander between this ambiguous ‘outsiderness’ and a propensity for integration as they navigate the multifaceted middle positions they occupy. Contextualising the findings in research on white migration movements from the West, the conclusion suggests that the class-based notion of middling migration should be refined by including economic, social and cultural ways of being ‘of the middle’.

## Introduction

Along with its transformation into the world’s second⁠ largest economy, China has become a new destination for international migrants (Pieke, 2012). In 2010, the Chinese national census counted 600,000 resident immigrants (Hugo, 2014). More recent data give a total of 900,000 foreigners working in China in 2016 (Huang and Yan, 2018) and the United Nations Population Division (2019) estimates that there were 1,030,871 international migrants in China in 2019. While they consisted mainly of Western corporate expatriates and diplomats in the early reform era, the diversity of foreign professionals has steadily increased, and now includes migrants who have come to China on their own initiative and have been hired locally by international and Chinese companies and entrepreneurs (Camenisch and Suter, 2019) from various geographical origins. Most foreigners come from nearby Asian countries, but ‘Westerners’ still constitute another major group, and are mainly US American, Canadian, French and German (Zhou and Elsinga, 2015).

Previous studies of the experiences of Westerners in China have been mostly conducted in a single city, such as Shanghai (Farrer, 2019; Stanley 2013), or Xiamen (Lehmann, 2014), or are focused on specific professional groups like English (Leonard, 2019; Stanley, 2013) or international teachers (Poole, 2019), or they have investigated the specific experiences of US Americans (Liu and Dervin, 2020). This article examines how Europeans are positioned and how they navigate their position against shifts in the dynamic, now they are no longer granted automatic elite status as white Westerners in China. The article is based on ethnographic research on middle-class migrant professionals from Switzerland who are, in many respects, representative of ‘middle-class, white, educated north-western Europeans' (Camenisch and Suter, 2019: 210). In the context of China, they are a subgroup of what Leonard and Lehmann (2019: 2) have framed as the ‘new wave’ of immigrants; Western foreigners who vary more greatly in their migration trajectory, occupation and age, and socio-economic position than the hitherto dominant group of corporate expatriates. As my interviewees, furthermore, live in eight different Chinese cities, I am thus able to describe the broad non-city or profession-specific patterns of European migrants’ positionalities.

This analysis of the multifaceted and shifting role of whiteness as a racialised and contested marker of a minority position in a non-Western country provides an empirically grounded impetus to overcome the Euro-American bias in critical whiteness research. It also attends to specific meanings attached to the national background of the migrants in China, whereas previous research has often focused on a rather generic group of Westerners. By applying the notion of middling migration to European migration in a predominantly non-white and rapidly developing Asian society, this article further aims to ground the – somewhat vague – notion of this term in empirical research and discusses possible ways in which it can be refined.

This article consists of six sections. The next section introduces the methodology and how critical whiteness studies, and the theoretical notions of middling migration and of translocational positionalities are employed in this article. The following section (‘othering' along racial and ethnic boundaries) discusses the crucial role of racial categorisation for how European migrants are positioned in China. Next, I discuss white foreignness positionalities; I unpack the way in which they navigate their white foreignness and I trace the continuities and ambiguities of white privilege, that are declining, however, in favour of a ‘Chinese ascendancy’. The following section, nuanced understandings of white middling positions, analyses the social boundaries and categories that interviewees refer to, create, reinforce or contest to position themselves as the ‘golden mean’ of Western foreigners in China. Finally, in the concluding section, I propose to develop the concept of middling migration to attend to class as well as to the gradual changes and degrees to which the positions of white European middle-class migrants may be shaped by coexisting racialised privilege and marginalisation and various economic, social and cultural ways of being ‘of the middle’.

## The Translocational Positionalities of White Swiss Middling Migrants in China: Theory and Methods

This article draws on 16 months of anthropological fieldwork in 2014–2015 during which I investigated the trajectories, aspirations and positionalities of Swiss professionals in mainland China – a small but rapidly growing group of foreigners from a European country with long-standing relations with China. Reflecting the increase of international migration to China, the Swiss officially residing in China nearly doubled between 1993 and 2018 to reach 3400 individuals (Bundesamt für Statistik, 2019). However, one-third of the Swiss people I interviewed have retained their residence in Switzerland and are not included in these statistics, which therefore probably underrepresent the actual numbers. Most of those who are registered on the mainland live in the consular regions of Shanghai, Beijing and Guangzhou (Bundesamt für Statistik, 2019).

Reflecting this spatial distribution of the group under study and the sampling strategy described below, my fieldwork was conducted in Shanghai, Suzhou and Hangzhou, Beijing, and four cities of the Pearl River Delta (Guangzhou, Shenzhen, Foshan, Zhongshan). The data collected comprise narrative interviews with 22 Swiss men and 11 women living in China, as well as numerous participant observations and informal exchanges with Chinese and foreigners of various origins. Sampling and analysis evolved following the method of constructivist grounded theory (Charmaz, 2006). Potential interviewees were identified by snowball sampling, by online research and on digital platforms and theoretically sampled to initially obtain a comprehensive picture of the study group and later to test and differentiate the key findings that emerged as the coding of data became gradually more refined and theorised. This paper mainly draws on 11 (out of 42) codes related to interviewees' positionality and social network.

The participants have grown up in, or spent the formative period of their lives in, Switzerland before moving to China. In view of the high proportion of foreigners (25%) in the Swiss resident population (Bundesamt für Statistik, 2020), Swiss nationality was not a sampling criterion. One respondent is a German and another a Spanish–Italian citizen, while the others are Swiss nationals, sometimes with a second citizenship. Respondents thus share a Swiss middle-class background but show great diversity in the duration of their stay in China, their age, occupation, and family situation. They were between 23 and 64 years old, mostly in their 30s and early 40s. Most hold a university degree and, apart from two corporate expatriates, they had migrated to China, or remained there of their own accord, ranging from young interns and small-scale entrepreneurs to established professionals. The duration of their stay varied between six months and 25 years. Most of my interviewees had been living in China for an extended period (8 years on average) and 15 (three women and 12 men) were in a relationship with a Chinese person.

I am a female, white Swiss anthropologist specialising in migration with a long-standing research interest in China. When I conducted fieldwork in 2014–2015, I was based in Guangzhou. I was in my late 30s and accompanied by my husband who taught temporarily at an international school, and our three children who were enrolled at the same school. Thus, I embodied a variety of characteristics – in terms of education, age, biographical stage and family status, nationality, race, professional trajectory, social environment and lifestyle in China – which led to a fieldwork constellation of studying 'sideways' (Hannerz, 2006) and enabled me to also reflect on the positionalities of foreigners autoethnographically.

In light of their middle-class status in Switzerland, I conceive of the transnational trajectories of the interviewees as a form of middling migration. Conradson and Latham (2005: 229) have introduced this notion to describe migrants with a ‘middling status position in their countries of origin. [...] they appear to be simply middle class. As to the societies they come from and those they are travelling to, they are very much of the middle’. This terminology has been taken up by several other authors (e.g. Ho, 2011) to investigate the diverse experiences of middle-class migrants from various national backgrounds at different destinations. I support Yang (2020: 2) in arguing that this research has often not adapted a sufficiently locally embedded perspective and that more empirically and contextually grounded research is needed to substantiate this ‘broad and arguably imprecise descriptive category’.

With specific attention to the role of racialisation and whiteness, this article suggests possible ways of deepening the understanding of this ‘being of the middle’. It thereby contributes to the literature at the intersection of critical whiteness studies and middling migration. While cautious of the global legacy of supremacy, normativity and privilege linked to whiteness, I build on Winddance Twine and Gallagher (2008: 6) ‘third wave’ of critical whiteness studies and approach whiteness as a socially constructed ‘multiplicity of identities’. I aim to understand how the positionalities of European foreigners categorised as ‘white’ in China are contextually and ‘historically grounded, class specific [...] social locations’ (Winddance Twine and Gallagher, 2008: 6).

To understand how notions of whiteness and other characteristics intervene in the positioning and incorporation of white, middle-class, Swiss migrant professionals in China, I employ the theoretical lens of translocational positionality (Anthias, 2002, 2012) and social boundaries (Barth, 1969; Wimmer, 2008). Anthias (2002, 2012) characterises translocational positionalities as the intersections between the positions that an individual is assigned in a specific structure and their own simultaneous agentic positioning as they strive to fit into or challenge these positions. This space and the positions in it are shaped by social boundaries along lines of perceived difference or similarity, often tied to markers such as race and nationality. These boundaries (Barth, 1969; Wimmer, 2008) delineate social categories from each other and work to ‘signal membership and exclusion’ (Barth, 1969: 15). Social categories are interactionally, situationally and contextually created and renegotiated and thus subject to ongoing boundary work (Gieryn, 1983) of self- and external positioning. Yet they also contain expressions of social hierarchies as vertical vectors of global dimensions of power (Anthias, 2012; Wimmer, 2008).

## ‘Othering’ along Racial and Ethnic Boundaries: Becoming a White Foreigner in China

‘One always remains a laowai^1^ (老外), a foreigner. […] As long as you have a “long nose”, that’s what you are’ (Anton, 55, sinologist and business consultant, several stays in China since 1983, living in Beijing since 2008).

### Race and ethnicity as markers of social boundaries

This example illustrates a general perception among my interviewees that their physical features play a fundamental role in their general social position in China which is primarily influenced by the prevalent distinction between Chinese as insiders and foreigners as outsiders. Like Anton, all but two participants can be characterised as ‘Caucasians’. In Switzerland, they belonged to the invisible racial majority (McIntosh, 1988) and then became ‘white’ by migrating to China and instantly marked as non-Chinese (Liu and Dervin, 2020).

Thus, following Murji and Solomos (2005: 1) understanding of racialisation as ‘processes by which ideas about race are constructed, come to be regarded as meaningful, and are acted upon’, physical markers take on the role of a racialised boundary. Looking ‘white’ acquires a specific meaning of socially constructed difference emerging during transnational migration from Europe to China.

This was also experienced in the opposite direction by the participants with an ‘East Asian’ appearance. Gary (23) is an ethnic Chinese who grew up in Switzerland and New Zealand and worked as an intern in Shenzhen. He told me that he is usually first taken for a local resident in China. Then, once people have become aware of his biography, they generally position Gary as a huaqiao (华侨), an ‘overseas Chinese’ rather than a ‘foreigner’. Didier (41), an entrepreneur in southern China for over 16 years, had a similar experience. Born in Korea, he was adopted by Swiss parents as a toddler and grew up in Switzerland as a, he said, ‘banana […] yellow outside, white inside.’ Didier explained that, due to his Asian features, he can to some degree cross the racialised social boundary that normally positions white foreigners as ‘others’:

I think that you white people can stay in China for 10 years, the integration will be different. […] [But] there is somehow a way that they look at an Asian, like a mirror, the image they receive from you is the same as who they are.

Since he lacks the markers of racial difference that distinguishes white foreigners, Didier is generally positioned in between the social categories of laowai and huaqiao.

Classifying foreigners on the basis of their whiteness usually coincides with ascribing to them a Western ethnicity and cultural background (Liu and Dervin, 2020) – before it is known that they are indeed from a country commonly seen as part of the West.^2^ Pieke (2012) explains this by pointing to the immigration trends to China in the 1970s and 1980s, when foreigners originated mostly from Western countries. Therefore, and despite the numerous Asians and white foreigners from countries usually perceived as non-Western such as Russia, ‘in the eyes of many Chinese, [white] Westerners are [still] the paradigm of a foreigner’ (Pieke, 2012: 47).

This racialised distinction has to be understood in the light of how the concept of race developed in China. According to Bonnett (1998), pre-modern white identities in China did not build on racialised notions. Rather, they were symbolic, used to distinguish between the presumably whiter-skinned Chinese elite from presumably darker-skinned, lower strata of society. As Bonnett (1998) further elaborates, the idea of the existence of, and distinction between, a yellow and a white race as an exclusively European identity was popularised in the West and then introduced into China.

In other (semi-) colonial contexts, racialised distinctions justified and established European and more in general white supremacy (McIntosh, 1988). In late imperial China, however, ‘the notion of a yellow race was a positive symbol of imperial nobility actively mobilised by reformers who transformed it into a powerful and effective means of identification’ (Dikötter, 1994: 410) that also contested the notion of European, and later, Western, white supremacy. When China became a republic in 1911, political thinkers such as Sun Yat-Sen accordingly thought of the Chinese nation as an ‘ethno-national community of biologically descendant Han Chinese, a racial state’ (Fennel, 2013: 251). After the foundation of the People’s Republic of China, this discourse was adapted under Mao from the 1950s–1970s when China was represented as ‘leader(s) of the victimised “coloured” people in the historical struggle against white imperialism’ (Dikötter, 1994: 191–192). However, the idea of white supremacy reappeared in the wake of China’s reintegration into the global economy since the 1980s and ‘many Chinese intellectuals understood the world according to an international racial hierarchy’ ranking the ‘white’ West higher than ‘yellow’ China (Lan, 2016: 304).

## Navigating Positionalities of White Foreignness

### Benefits and humiliations: the contradictions of (Swiss) white privilege

My participants agree that, as white European foreigners, their attributed status in China is unlike the one in Switzerland, where most interviewees described themselves as ‘normal’ members of the domestic (predominantly white) middle class. This position was often reformulated into a more conspicuous and generally higher social status in the wake of their transnational migration to China. This becomes manifest, for instance, in smaller or greater so-called ‘foreigner bonuses’ (see also Lan, 2011) such as preferential treatment in nightclubs, or customer services that they would not have received in Switzerland and which they also felt would not be given to a Chinese person with a comparable socio-economic background.

My interview partners explain these advantages by the generally still favourable perceptions of the West in China, and related projections of affluence, modernity and high quality on white foreigners. Thus, the local adaptions of historical continuities of postcolonial global power hierarchies, the long-standing economic dominance of Western countries as well as their discursive power to influence the parameters of aspired-to modernity (Meinhof, 2018), have created a privileged niche of social acceptability for white Europeans in China.

#### Reflecting on and exploiting Swiss racial privilege

Many added that these positive responses to their whiteness in their initial encounters in China are intersected and enhanced by their nationality. In their experience, when interviewees mention their national background, their Chinese interlocutors often react with describing Switzerland in positive stereotypical terms mentioning affluence, dependability, and high moral qualities (as well as chocolate and watches). Anton noted:

As a Swiss in China you have the advantage that Switzerland was, together with Sweden, the first Western country to recognise China. And therefore, members of the Chinese elite, especially, perceive Switzerland as a friendly country. Which means that we [Swiss] are more easily accepted than, for instance, someone from the States.

Bernie (42) a carpenter and product designer, who has lived in Shanghai for 9 years and works as an art teacher, compared his position as a Western, Swiss foreigner with that of his Filipina wife, alluding to the comparably lower place South Asians are located in the ‘racial hierarchy’ (Lan, 2016) of foreigners in China:

Well, as a Filipina, Jenny is certainly labelled as being in a much lower bracket than me. Not in an upper bracket where you can tell Chinese people to do this and that, show them how to develop and invent something.

At the same time, most of the participants also exploited the privileges inherent in their position to some degree. They navigated their foreigner status in business by emphasising the markers that benefitted them in a specific context. One example of this is Brian, an entrepreneur, sinologist and British–Swiss dual citizen in his mid-twenties. After participating successfully in a popular Chinese-language TV competition and achieving a certain popularity, he first presented (in Chinese) and directed TV documentaries and then launched a start-up company for air purification. His command of Chinese, and his familiarity with China from a Western perspective were central to his professional positionality during his years in television. However, he started to emphasise his Swiss origin when he became an entrepreneur:

It’s useful because Switzerland is one of the best brands in the world. It made no difference when I was making documentaries. But when I’m trying to sell a health product, then, the fact that the product is from Switzerland helps. And the filters inside actually are from Switzerland, so that’s good. […] And then, of course, there are a lot of fake foreign products in China. […] But if you have an actual Swiss person saying: ‘So, this is our product from Switzerland’ – OK, no question, it’s real.

Some interviewees also reflected on how they had, at some point, normalised the foreigner bonus and started to take their ascribed white supremacy for granted. They interpreted this change as a deviation, in the sense that it had led them to violate their original Swiss middle-class understanding of respectful social interaction. Derek, who had previously lived in Malaysia and then moved to Dongguan, noted that he had changed during his stay in Malaysia:

In the beginning, you look at people [Westerners] who have been there for a long time and you say: ‘These people are assholes’. And then you start to behave like them. […] When you go out, sometimes you are a bit disrespectful. You go over the limits […] I remember, one moment very well: I was in a five-star hotel. I always stayed there; the staff knew me. And there was this guy, working in the hotel. […] He looked straight into my eyes and he said: ‘Who do you think you are?’ […] And I really believe that I am a respectful person. […] And at that moment, I thought: ‘OK, so, I have to be careful now because I’m becoming an asshole myself’.

#### Mobilising whiteness in unequal bargains

However, the implications of social status boosted by notions of a national-specific white privilege can also lead to disadvantageous, even humiliating, situations. Many interview partners realised that some of the professional and social opportunities that were open to them as a result of their whiteness and foreignness were, in fact, double-edged swords. For instance, they may be employed by a company because they have a foreign, or white face – a phenomenon that Stanley (2013) described in the field of English teaching which reaches its most pronounced form in ‘rent-a-foreigner’ gigs (Borenstein, 2016). While such positions offer the opportunity to convert whiteness into economic capital, they often come without relevant responsibility and thus their incumbents lack credibility and social acceptance as professionals (Lan, 2011).

This happened to Bernie, who talked about his job in a preparatory art school for Chinese high school students. ‘On my name card, it says “Academic Director”. What it really means, is “foreign face”. I think that’s more or less it’. Being employed as the white face that represents the school’s international character, while not being allowed to develop the international branch’s curriculum of which he was officially the academic director, results in a feeling of humiliation, powerlessness and uselessness at work.

Many participants also noted that they felt they were being used by some of their Chinese acquaintances for the same reason. Hugo (50) who has been living as an entrepreneur and restaurant owner in Shenzhen for 5 years, remembered:

In the first two years I was very often invited to various events (by Chinese acquaintances). But in fact […] the people who invited me did so to show that they knew a foreigner. […] And that makes you feel exploited.

The interviewees’ expectations of their friendships include a mutual interest in each other’s personality and well-being. However, they found that this did not apply to some of the social relations they had with Chinese individuals which they had initially interpreted as friendships. What they had believed was a relationship of mutual emotional comfort seemed to serve these Chinese ‘friends’ primarily as a means of gaining cosmopolitan capital and ‘face’ in the eyes of other Chinese (Liu and Dervin, 2020) and was thus experienced as an unequal bargaining of resources.

### Fading white supremacy versus Chinese ascendancy

The existence of the white foreigner bonus and social and professional benefits and opportunities (often enhanced by the positive image of Switzerland) they bestowed on the positionalities of Swiss migrant professionals in China can be read as expressions of white privilege (McIntosh, 1988), as a mobile global resource. This China-specific reformulation of white privilege seems thus embedded in a racialised order that is still greatly influenced by postcolonial narratives of a global white supremacy (Lan, 2016; Lehmann, 2014; Leonard, 2010) linked to varied imaginaries of ‘Western modernity’ (Meinhof, 2018).

Yet this continued, and ambiguously experienced, ascription of white supremacy is only one side of the multifaceted meanings attached to racial whiteness in China. As Bonnett (2018: 1201–1202) has argued, ‘an exclusive focus on race as a Western “invention” is inadequate’ and that, while remaining attentive to the undisputedly profound Western influences, racialisations should be approached as varied and interwoven with equally multiple projects of modernity. Since the 1980s, both Chinese society and Western immigrants have diversified greatly and nowadays migrants engage more closely with various local populations (Pieke, 2012). This has contributed to an increasingly differentiated, situational and more interactive racialisation (Lan, 2016) of whiteness. Accordingly, many participants observed how, along with China’s rise to become a global power and the increase in the level of education and standard of living of many Chinese, Western foreigners are gradually losing their aura of supremacy and attractiveness, especially among urban populations and domestic elites.

Due to the analytical focus on the interplay of middling migration, whiteness and nationality and lack of space, gendered aspects are not further discussed in this article. However, it should be mentioned that the observed development is also expressed in the gender relations experienced by my interviewees. An affluent Chineseness seems to be gaining ground against a less positive image of Western masculinity (Farrer, 2011) and many male respondents pointed out that their earlier attractiveness to Chinese women has diminished greatly. Hoang (2015: 10) has similarly noted that intimate gender relations in the Vietnamese global sex industry are increasingly marked by an Asian ascendancy and a simultaneous Western decline, induced by the economic rise of South-East and East Asia, which has fostered new images of pathways ‘toward modern nationhood with respect to Asia and the West’. Interviewee Max (31) emphasised that, especially in the eyes of Chinese women from a privileged urban background, white masculinity has lost its former connotations of affluence and cosmopolitanism. He has been living in Beijing for 12 years, first as a student of the Beijing Film Academy and then as a freelance professional in the film industry, noting:

When I first came to Beijing […] I would go out and come across at least one or two girls who would smile at me and think: ‘Wow, this Westerner is really cool’. Nowadays […] Westerners in China don’t have that much money in comparison with some Chinese. Foreigners used to be perceived as more sophisticated, and who had money and treated women much better than locals. Foreign men were perceived as a better choice. Nowadays, except for those girls who really want a foreigner as a boyfriend, most prefer a rich Chinese.

The meanings attached to whiteness as they are reformulated and renegotiated in the Chinese context are hence marked by synchronous privilege, marginalisation and loss of status. Even though the specific reconfigurations of whiteness in China still produce limited racial privileges, they are equally shaped by the resurgent tradition of meeting the presence of foreigners, foreign institutions and foreign investment with suspicion and of controlling their presence to make ‘the foreign serve China’ (Brady, 2003). The symbolism of white privilege that lingers on in China is thus not grounded in a powerful, structural white supremacy. The role of Western foreigners resembles that of ‘the foreign support-staff for the Chinese Dream’ more than that of postcolonial ‘power migrants’ (Farrer, 2019: 99–200). Although whiteness is generally conceptualised in terms of having agency (Garner, 2017), my findings illustrate that Western foreigners in China are not the ones who determine the racial order and the attributions and opportunities related to their whiteness. Rather, against the background of shifting power asymmetries and related multiplying racialisations (Bonnett, 2018), they become the visible white foreign ‘other’ and the object of an increasingly ambiguous ‘Chinese gaze’. The position of whiteness at the top of global power hierarchies and the dominant status of Western modernity are thereby challenged and contradicted by nationalist and racialised counternarratives of Chinese supremacy (Cheng, 2019).

Therefore, rather than taking experiences of white privilege as a manifestation of enduring and institutionalised white supremacy, in contemporary China it should be read as ‘white supremacy outside, Chinese ascendancy inside’. In places where whiteness is evoked and used as a resource, this seems to favour Western foreigners’ positionalities to some extent, but it also benefits their Chinese interlocutors.

## Towards a More Nuanced Understanding of White Middling Positions in China

Studies of Western migrants in non-Western locations tend to portray them as transnational elites (Beaverstock, 2002), and have foregrounded the postcolonial continuities in white migrants’ positions (Fechter and Walsh, 2010; Leonard, 2010) entailing their self-segregation in economically and socially privileged ‘expat bubbles’ (Fechter, 2007; Lundström, 2014). Partly challenging this stereotype, recent research in China points to the diminishing and restricted privileges of Western foreigners in China, noting at the same time, however, that they nevertheless occupy the segregated position of the white ‘other’ (Leonard, 2019; Liu and Dervin, 2020).

### The possibilities and limitations of integration

Yet, expanding on Farrer (2019) findings for Shanghai, the narratives of my own interviewees express more inclusive ways of self-positioning and of dealing with their (ascribed) foreign white otherness. Many challenge their categorisation as a laowei in terms of its implication that they do not belong in China, as film director Max (34) said:

Yesterday I had this business meeting with this Chinese guy. […] And he made me feel like: ‘You’re a foreigner, you’re a foreigner. You will never know anything as well as a Chinese does, you will never be able to experience something the way a local does. Never! […] You will never be a Beijinger’.

However, Max fervently contested this ‘othering’ arguing that: ‘I mean, probably I have been living in Beijing for longer [12 years] than this guy’ and noting that he has received his university education and most of his professional experience in China, and has a Chinese significant other.

As I have elaborated in another article (Camenisch, 2019), the approach of the interviewed migrant professionals to living in China is framed by their aspiration of challenging and developing themselves through immersion. An ideal they frequently evoke is that of integration, expressing their willingness to adapt culturally and be incorporated in the social realm. Accordingly, many interview partners said that their aim was to ‘live among Chinese people’ and to become ‘integrated’. Gregor, a middle-aged and locally employed high-ranking manager in a European transnational company, having lived in several Chinese cities since 1992, told me:

I’m quite well integrated here. I’m not at all like those foreigners who live in a bubble and have no contact with local people at all. [...] And I live in a housing complex in which at least half of the inhabitants are local […]. Most of my friends are local [Chinese]. […] I think I behave quite normally. And I don’t lead an expat lifestyle like you sometimes see. […] They only eat and buy Western stuff and complain about everything. […] And after years of living here, they still don’t speak a word of Chinese. […] But these are those foreigners who don’t stay here for long. Those who have stayed for a long time [like him], are a different breed.

Like Max and Gregor, both the male and female interview partners presented themselves as embedded in, and knowledgeable about, their Chinese environment. Many speak Chinese fluently or proficiently and some have received all or a part of their university education in China. They share an interest in China’s history, culture and current affairs – and consciously try to adapt to what they perceive as local ways of life. More than half of my subjects have a Chinese significant other and many among them have started a family and thus have Chinese relatives and binational children. This adds another layer to the various ways in which many interviewees feel connected to, and are entangled with, their host society and adds a pronounced degree of permeability and incorporation to their complex outsider positionality.

This (striving for) gradual entanglement also extends to the sphere of socialising. On the one hand, as elaborated above, establishing close friendships with Chinese was experienced as a rather rocky territory by most, and is explained by perceived cultural differences in socialising (Camenisch and Suter, 2020). On the other hand, like Gregor, most interviewees proudly emphasised that they had Chinese friends. However, many felt that their positionality as foreigner permeated their social relations with Chinese and often still assigned to them a subordinate position, especially in a hierarchical constellation or when a conflict may arise.

Thus, the interviewees who had lived in China for many years reflected that if they have developed close and equal relationships with Chinese, it was with people whom they saw in one way or the other in a similarly marginal position as ‘Chinese outsiders’. Charlotte, a transnational entrepreneur in her late 30s who had been living in Beijing for 7 years reflected, for instance:

I have very few Chinese friends. And those I have are somehow atypical. One is gay, the second is a Catholic, and the third is married to an American. They are all people who do not fit into the system.

### Internal boundary work: Claiming the ‘golden mean’ of foreignness

Gregor’s statement points to two other facets of my interviewees’ active positioning themselves as being closer to the heterogeneous Chinese society than other foreigners are.

Firstly, most of them said their class position was similar to that of many (upper) middle-class urban Chinese and was considerably more modest than that of wealthy foreigners in China and Chinese elites. Corporate expatriates were often mentioned in this boundary work and described as the foreigners with the highest salaries and most ‘de-localised’ lifestyles. The way of being ‘of the middle’ also is connected to the professional incorporation of these migrants in China. Unlike the corporate expatriates who work for, and in the interests of, Western transnational corporations, the economic activities of these Swiss entrepreneurs and locally engaged staff primarily address the domestic market and rely upon the purchasing power and changing lifestyles of the Chinese middle and upper classes.

Secondly, the interview partners distanced themselves from other foreigners by positioning themselves on ‘the golden mean’ of a spectrum. On the one hand, many stressed that they are better educated and have more responsible jobs or more serious enterprises than foreigners who are more economically and socially marginalised. This includes Westerners in low-prestige professions in which they mainly capitalise on their racial and ethnic appearance rather than on professional skills – specifically English teachers who have become a stigmatised group of white ‘low-quality foreigners’ (Leonard, 2019: 151) in China. In Didier's words:

The reality is, you’ve got really two breeds of foreigners in China. You have got the real entrepreneurs, their focus is fixed, they know what is going to work. […] Then you got […] English teachers […] they are losers. […] And there are some claiming they are entrepreneurs but they’re actually losers and drifters [...]. They are here because the benchmark in a lot of industries is so low.

On the other hand (corporate) expatriates are portrayed as often arrogant, consciously self-segregating and uninformed about China who thus possess some of the characteristics that the traditional expatriate research has ascribed to Western migrants. Therefore, and because both the scholarly as well as the colloquial usage of the term expatriate is ‘usually and intuitively reserved for [white] Western migrants’ in general (Kunz, 2016: 91), these European professionals reject identification with typical ‘expats’. Instead, their boundary work aims at carving out a differently characterised, more middling and integration-oriented niche in the broader category of ‘foreigners’ in China, as Véronique said:

I would say, I’m a happy, integrated foreigner in China. [...] Because I am still […] not a local. [...] I’m quite tired of the expats. [...] Because as a foreigner living in China, I think, it’s important to integrate. Or to look at Chinese as equals’.

The images these interviewees evoked when describing their positionalities as foreigners in China and in relation to their Swiss origin are often those of the in-between, but also of the bridge, or of the middle. In the social sphere, this middling position is not only expressed by the existence of friendships and intimate relations with Chinese (outsiders), but also through a prevalence of friends and significant others – both Chinese and foreigners – with similar experiences. Gregor told me about his Chinese wife: ‘My wife is Chinese but she […] went to school in England. I guess she’s culturally as much in-between as I am. Therefore, it works well’. And with regard to friendships, Brian noted:

I don’t have any traditional expat friends because I find them frustrating. We’re in a very different culture now. [...] half the time, I almost speak Chinglish, there are Chinese words in my English. And with my friends, we do that all the time. […] It’s hard in China to find people like yourself. Because you have got expats, foreigners who come here for one, two years. They don’t understand China. And then, Chinese people don’t understand the Western way of thinking. And I feel very in the middle.

Positioning oneself as ‘in the middle’ between Switzerland, or, more generally, ‘the West’ and China as well as on ‘the golden mean’ among Western foreigners in China thus serves to underline varying degrees of cultural, social and economic embeddedness and entanglement in their Chinese contexts as a simultaneously still ‘othered’ white European. The analytical reflection of such representations of a multidimensional way of being of the middle adds a further nuance to hitherto class-related understandings of middling migration.

## Synthesis

Altogether, this article has described how Europeans are treated as different in their everyday encounters in China due to their phenotypical characteristics, which are often automatically characterised as being of an ethnic Western origin and racial whiteness and starkly contrasted with the cultural and racial concept of being ‘Chinese’. Nationality is, then, a further attribute that influences their ascribed position within the broader social category of ‘foreigner’.

As they become white, Western ‘others’ in China, many Swiss professionals experience a slight degree of upward mobility and the benefits that flow from this. This is rooted in a re-emergence of white privilege during the period of Chinese reform and the related preference for values associated with Western modernity. However, the racial hierarchy in China is multi-layered, contested and in a state of flux, and white foreigners are exposed to intertwined processes of privilege, inclusion and marginalisation. A racialised Chinese nationalism, China’s global rise and the related emergence of a Chinese middle and upper class have been seen as factors leading to the reduction of a pre-existing privileged whiteness in China. The ways white privilege is evoked in Chinese contexts may produce limited benefits to many foreigners but may also entail disadvantages and humiliation. What may at first sight look like manifestations of enduring white supremacy are therefore its subjugated vestiges, devoid of real institutional power and without agency in the racial hierarchy. If they are reassembled, this merely signals Chinese ascendancy and racial equality, if not superiority, in the first place.

At the same time, the interviewed Europeans actively contribute to renegotiating the shifting, multifaceted and situational social boundaries around their white foreignness and strive for a reflexive and differentiated way of living within Chinese society. In claiming to be the golden mean, such middling migrants seek to create a new niche within the category of the white ‘other’, marked by a pronounced degree of permeability and incorporation. This is also manifest in the ways many interviewees share feelings of being somewhere ‘in the middle’ of a Chinese and a Western way of thinking and living.

On the conceptual level, this article has demonstrated that the positionalities of Swiss migrant professionals in China are marked, not only by their whiteness and their transnational middle-class mobility, but also by additional forms of occupying a middling position. I therefore suggest that, in a further move away from traditional expat scholarship, research on European – and more generally, Western – migration must enrich the notion of middling migration. While remaining attentive to the nuanced and context-specific continuities and contestations of white privilege, further theorisation should not only address Western migrants’ class background but also the degree of their economic, social and cultural incorporation in their destinations as well as the gendered nature of their positions. The notion of translocational positionalities has proved fruitful for unpacking the various social boundaries marking their multi-layered middling positions and to understand how they are negotiated and reformulated by foreigners and Chinese citizens in stratified, situational and dynamic boundary work.

The Chinese state and society are only hesitantly adjusting to the reality of immigrants in their midst. Yet the growing presence of long-term foreign residents, as well as new generations of people with a mixed Chinese-foreign background, emphasises the need to reconsider social and legal boundaries between foreign outsiders and Chinese insiders. As the positions of Western middle-class migrants develop in the context of Asian ascendancy and a racialised Chinese nationalism, the meanings of their whiteness and foreignness will be further contested and refashioned. Against this background, it will be interesting to see whether political and social responses to the current COVID-19 pandemic will entail the creation of new boundaries and the renegotiation of existing ones for white foreigners in China.

## Notes

1. Other common ways of labelling a foreigner are the more formal ‘waiguoren’ (foreigner) or ‘waiguopengyou’ (foreign friend). The point here is not to explore the specific connotations of these terms but to note that they all refer to and maintain a social boundary that distinguishes ethnic Chinese from (white) foreigners.

2. I employ the term here in a colloquial sense to refer to western Europe and northern America as well as Australia and New Zealand, without implying any analytical essentialism.
